# Rethinking urinary antibiotic breakpoints: analysis of urinary antibiotic concentrations to treat multidrug resistant organisms

**DOI:** 10.1186/s13104-018-3599-8

**Published:** 2018-07-20

**Authors:** Daniel B. Chastain, S. Travis King, Kayla R. Stover

**Affiliations:** 1 University of Georgia College of Pharmacy, 1000 Jefferson Street, Albany, GA 31701 USA; 2 Ochsner Medical Center-New Orleans, New Orleans, LA 70121 USA; 3 University of Mississippi School of Pharmacy, Jackson, MS 39216 USA; 4 Division of Infectious Diseases, University of Mississippi Medical Center, Jackson, MS 39216 USA

**Keywords:** Urinary tract infections, Multi-drug resistant, Antibiotic, Urine concentration, Pharmacokinetics, Pharmacodynamics

## Abstract

**Objective:**

The present study analyzed whether renally eliminated antibiotics achieve sufficient urinary concentrations based on their pharmacokinetic/pharmacodynamic principles to effectively eradicate organisms deemed resistant by automated susceptibility testing.

**Results:**

Lower median minimum inhibitory concentrations against enterobacteriaceae were noted for ceftriaxone, cefepime, and doripenem when comparing Etest^®^ to Vitek^®^. All *Pseudomonas aeruginosa* isolates were susceptible to cefepime, ciprofloxacin, and doripenem with both susceptibility methods, but higher median minimum inhibitory concentrations were observed with Etest^®^. Urine concentrations/time profiles were calculated for standard doses of ceftriaxone, cefepime, doripenem, and ciprofloxacin. The data presented in the current study suggests high urine concentrations of antibiotics may effectively eradicate bacteria which were determined to be resistant per in vitro susceptibility testing.

**Electronic supplementary material:**

The online version of this article (10.1186/s13104-018-3599-8) contains supplementary material, which is available to authorized users.

## Introduction

Infection with multidrug-resistant organisms (MDROs) commonly requires treatment with new or investigational compounds, or alternatively, older or even potentially more toxic drugs. New antibiotics are greatly needed to treat infections caused by these pathogens, primarily Gram-negative bacilli [1, 2]. Development of novel antibiotics represents the most attractive solution against emerging resistance; however, a more immediate solution is the strategic repurposing of older or more narrow-spectrum antibiotics [3–5].

Clinical and Laboratory Standards Institute (CLSI) publishes consensus standards and guidelines annually, which include recommendations for minimum inhibitory concentrations (MICs) for various organisms against a wide array of antibiotics [6]. The in vitro susceptibility breakpoints recommended and reported are based on known serum concentrations of antibiotics, regardless of the anatomical site of infection. In 2014, CLSI created a urine-specific MIC for cefazolin against enterobacteriaceae (susceptible ≤ 16 µg/mL) [3]. Additionally, cefazolin susceptibility may be extrapolated to oral cephalosporins for the treatment of uncomplicated urinary tract infections (UTIs) caused by *Escherichia coli*, *Klebsiella pneumoniae*, or *Proteus mirabilis*. Modification of these breakpoints was likely the result of increased understanding of pharmacokinetics (PK)/pharmacodynamics (PD) as a predictor of efficacy.

Treating UTIs is based on achieving adequate antibiotic urinary concentrations in relation to the susceptibility of the offending pathogen, although this only represents a minor piece of the puzzle [7, 8]. Susceptibility results are unable to account for the crucial principle of PK, which is ultimately the time course of drug in tissues or fluids. Previous studies have proven the ability to eradicate bacteria from urine is dependent exclusively on urine drug concentration [7, 9, 10]. Glomerular filtration serves as the primary mechanism regulating the concentration of antibiotic that reaches the tubular lumen, while tubular secretion acts as a secondary excretory route for certain antibiotics [8]. A higher molecular weight and greater extent of protein binding further limits the amount of antibiotic that is filtered. The rate and extent of renal elimination of aminoglycosides and sulfonamides correlate with renal function as they are solely excreted by glomerular filtration. In contrast, penicillins, cephalosporins, and fluoroquinolones are eliminated through both glomerular filtration and tubular secretion, allowing for high urinary concentrations.

Increasing rates of MDROs limit effective treatment options, and limited studies exist evaluating the outcomes of treating UTIs with an antibiotic to which the infecting organism is resistant in vitro [11]. However, significant emphasis has been placed on optimizing antibiotic administration based on PK/PD principles, and it is now recommended in guidelines from the Infectious Diseases Society of America [12, 13]. The purpose of this study was to determine whether renally eliminated antibiotics achieve sufficient urinary concentrations based on their PK/PD principles to effectively eradicate organisms deemed resistant by automated susceptibility testing (AST).

## Main text

### Materials and methods

In this in vitro susceptibility analysis, multidrug-resistant Gram-negative bacilli, defined as those resistant to at least 1 agent from at least three classes of antibiotics, were identified through a Vitek^®^ susceptibility report at an academic medical center and tertiary referral center. Isolates obtained from urine cultures in both critically ill and ward patients 18 years of age or older admitted from November 2013 to April 2014 were included. Patient demographics were not collected. Four antibiotics, including ceftriaxone, cefepime, ciprofloxacin, and doripenem, were chosen based on previously published urinary/time concentration profiles, which detailed specific urine concentrations based on time from prior dose, in healthy individuals [14–17]. MICs for each antibiotic were determined using Vitek^®^ and Etest^®^ methodology [18, 19]. Briefly, a bacterial suspension was made by selecting 1–2 fresh colonies and mixing it with 3 mL of sterile water to yield a suspension equivalent to McFarland Standard 0.5. Mueller–Hinton II Agar was then inoculated with the bacterial suspension to create a lawn. Etest^®^ strips for each antibiotic were then applied to the MHA, and the plates were incubated for 16–20 h at 35 °C. After incubation, the susceptibility of each organism was determined and verified by a clinical microbiology technologist. All procedures followed CLSI protocols, and MICs were interpreted according to CLSI breakpoints [18, 19].

Analysis was performed by comparing published literature detailing urinary antibiotic concentration/time profiles with in vitro susceptibility testing, through MICs, to determine if concentrations were sufficient to achieve the PK/PD target. For cephalosporins and carbapenems, this was defined as achieving concentrations greater than 4–5× the MIC for at least half of the dosing interval [8]. The percentage of time concentrations remained above MIC (T > MIC) was calculated with the following equation:

$$T > MIC\;(\% ) = ln\frac{dose\;(mg)}{Vd\;\left( L \right)x\;MIC\;(mg/L)}\; \times \;\frac{t1/2\;(hrs)}{\ln (2)}\; \times \frac{100}{dosing\;interval\;(hrs)},$$

where the percentage of the dosing interval that drug concentration remains above the MIC is represented by T > MIC (%), dose is the amount of drug in milligrams, volume of distribution (Vd) is the apparent volume of distribution in liters, MIC is the minimum inhibitory concentration (mg/L), half-life is represented as t1/2 in hours, and DI is the dosing interval in hours [20]. For fluoroquinolones, the PK/PD target was identified as achieving a peak concentration (Cpk) to MIC ratio of at least 10 [8]. These values were compared and validated with Monte Carlo simulations [17, 21, 22]. Additionally, MIC_50_ and MIC_90_ were calculated for comparison of results obtained with Etest^®^ with those from Vitek^®^. Statistical analysis was performed with descriptive statistics.

### Results

A total of 24 unique organisms were obtained from the Clinical Microbiology Laboratory based on their Vitek^®^ susceptibility report. Microbiologic susceptibility testing was performed following standardized CLSI protocols and techniques [18, 19]. Twenty-one of the isolates were enterobacteriaceae, including *E*. *coli* (n = 10), *Enterobacter cloacae* (n = 3), *P*. *mirabilis* (n = 3), *Citrobacter* sp. (n = 2), *K*. *pneumoniae* (n = 1), *Serratia* sp. (n = 1), and *Morganella morganii* (n = 1), while only 3 were *Pseudomonas aeruginosa*. An Additional file 1: Table S1 summarizes in vitro Etest^®^ susceptibility distribution. Lower MIC_50_ against enterobacteriaceae, based on Etest^®^, was noted with ceftriaxone, cefepime, and doripenem, but not ciprofloxacin (Table 1). Among all enterobacteriaceae isolates, ciprofloxacin had the lowest susceptibility rates, 20 and 15%, with Etest^®^ and Vitek^®^, respectively. Doripenem resistance was identified using Etest^®^ in only one isolate, *C*. *freundii*, while resistance was observed with 100% of *E*. *cloacae* (n = 3) via Vitek^®^. All *P. aeruginosa* isolates were susceptible to cefepime, ciprofloxacin, and doripenem with both susceptibility methods; however, higher MIC_50_ were observed with Etest^®^ (Table 1).

**Table 1:** MIC_50_ and MIC_90_ of enterobacteriaceae (n = 21) and *Pseudomonas aeruginosa* (n = 3) obtained with Etest^®^ compared to Vitek^®^

Antibacterial agent	MIC_50_ (µg/mL)		MIC_90_ (µg/mL)	
	Etest^®^	Vitek^®^	Etest^®^	Vitek^®^
Enterobacteriaceae (n = 21)				
Ciprofloxacin	32	4	32	4
Ceftriaxone	0.16	1	256	64
Cefepime	0.19	1	42	28
Doripenem	0.047	0.25	1.9	16
*Pseudomonas aeruginosa* (n = 3)				
Ciprofloxacin	0.125	0.25	–	–
Cefepime	2	1	–	–
Doripenem	0.25	0.5	–	–

T > MIC for at least 50% of the dosing interval was calculated for ceftriaxone, cefepime, and doripenem using the aforementioned equation and previously published PK values [14, 23, 24]. Standard ceftriaxone dosed at 1000 mg daily achieved T > MIC for > 24 h with an MIC of 4 mg/L. Cefepime was able to provide adequate T > MIC for ≥ 8 h against MIC of 8 and 16 mg/L with 1000 and 2000 mg every 8 h, respectively. Doripenem 500 mg every 8 h maintained T > MIC for > 8 h with an MIC of 4 mg/L. Ciprofloxacin 500 mg every 12 h, orally, was associated with a Cpk:MIC ratio of > 10 with an MIC of 32 mg/L [25]. Urine concentrations/time profiles corresponding with the above doses are much greater than those isolated from serum in healthy volunteers (Table 2).

**Table 2:** Average serum and urine concentrations following single dose administration in healthy volunteers

Antibacterial agent	Serum		Urine	
	Cpk (mg/L)	Ctr (mg/L)	Cpk (mg/L)	Ctr (mg/L)
Ceftriaxone 1000 mg IV [14]	151	ND at 24 h	995	ND at 24 h
Cefepime 250 mg IV [15]	17.9	0.6 at 8 h	190	90.2 at 8 h
Cefepime 1000 mg IV [15]	65.1	2.7 at 8 h	–	–
Ciprofloxacin 500 mg PO [16]	2.46	0.22 at 12 h	394	35 at 12 h
Doripenem 500 mg IV [17]	20.2	–	601	49.7 at 8 h

*Cpk* peak concentration, *Ctr* trough concentration, *hrs* hours, *ND* not determined

Discrepancies in interpreting susceptibility results of Vitek^®^ compared to Etest^®^ were discovered for 43% of organisms tested. More enterobacteriaceae (n = 2) were noted to be intermediate to cefepime with fewer susceptible and resistant isolates with Etest^®^ compared to Vitek^®^. Among the *E*. *cloacae* (n = 3) with documented resistance to doripenem via Vitek^®^, only one had intermediate resistance with an MIC of 2 mg/L while the remaining had MICs lower than the susceptible breakpoint with Etest^®^.

### Discussion

Antibiotic options are dwindling as the incidence of MDROs increases. Unfortunately, novel antibiotics are not being developed fast enough; therefore more reliance should be placed on repurposing antibiotics currently available. To the best of our knowledge, the present study is the first to examine comparative serum and urine concentrations of certain antibiotics in relation to PK/PD targets as a potential therapeutic intervention for UTIs, particularly uncomplicated UTIs, caused by MDROs. Although these values cannot be correlated with clinical efficacy and safety, it is important to recognize that a discrepancy between serum and urine breakpoints may exist. Urine concentrations that far exceed serum concentrations may be able to achieve appropriate PK/PD targets against organisms with increased MICs. Additionally, due to discrepancies in interpreting susceptibilities results between AST, such as Vitek^®^, and Etest^®^, interventions should also target increasing instrument accuracy.

Glomerular filtration and tubular secretion serve as excretory routes for most antibiotics [8]. High urine concentrations, sometimes 100- to 1000-times higher than those achieved in the serum with equivalent doses, occur as a result of these combined mechanisms. In vitro susceptibility breakpoints published by CLSI and reported by microbiology laboratories following AST are based on achievable serum concentrations rather than those obtained in urine [6, 8]. The clinical significance of this is not known. Recently, CLSI created a urine specific breakpoint for cefazolin, which is higher than those established for serum [3, 4]. This novel development represents significant advancement stemming from greater understanding of PK/PD.

While laboratory-confirmed antibiotic resistance is commonly associated with treatment failure, clinical response does not occur in all patients infected with a susceptible organism. In addition, those infected with an organism that is resistant in vitro to the antibiotic they receive do not always fail therapy [6]. Of the few studies that have analyzed clinical outcomes of patients with UTIs caused by resistant organisms, most are secondary analyses with small numbers of patients. Bacterial eradication was achieved in 50% of the 14 women assigned to trimethoprim/sulfamethoxazole (TMP/SMX) who had a TMP/SMX-resistant pathogen [26]. Additionally, 50% of the 10 women randomized to TMP/SMX with acute, uncomplicated, symptomatic UTIs caused by a TMP/SMX-resistant *E. coli* experienced bacterial eradication and clinical cure [27]. The rationale to explain this variability may lie in the susceptibility methods used and patient characteristics, but may be the result of increased urinary concentrations of TMP/SMX, previously associated with antibiotic efficacy in UTIs [8]. It is important to note that 25–42% of women with uncomplicated cystitis may resolve spontaneously; however, increased microbiologic cure and symptom resolution was observed with antibiotics [28]. Although the risk of progression to pyelonephritis and invasive disease is low, adherence to current guidelines is remarkably low and increasing isolation of MDROs may prompt clinicians to reconsider delaying initiation of antibiotics [29, 30].

Based on the results of our study and the achievable urinary concentrations from published literature compared with Monte Carlo simulations, we identified organisms with MICs that were determined to be resistant based on CLSI susceptibility breakpoints, but could potentially be eradicated with usual adult doses [8, 14, 17, 21–25]. Time-dependent antibiotics exert optimal bactericidal effects when drug concentrations are maintained above the MIC for at least 40–50% of the dosing interval. Concentration-dependent antibiotics achieve greater bacterial killing with increasing concentrations of drug, specifically, in the case of fluoroquinolones, when Cpk/MIC ratio is > 10. Comparing calculated serum PK/PD targets with those previously published, including Monte Carlo simulations, in association with serum and urine PK values may allow for more options against MDROs when antibiotics are warranted.

### Conclusion

Due to increasing rates of infections caused by MDROs, available treatment options are limited. The combination of previous data and the in vitro data presented in the current study suggests high urine concentrations of antibiotics may effectively eradicate bacteria which were determined to be resistant per in vitro susceptibility testing. The clinical implications of these results may be significant, allowing clinicians to forgo choosing more toxic antibiotics and instead select a more tolerable agent. An understanding of the PK/PD of these antibiotics is critical when applying this information, but additional data are needed prior to implementation into clinical practice.

## Limitations

The small sample size of urine cultures obtained based on results from their Vitek^®^ susceptibility report represents a limitation of our study. Additionally, when interpreting the results, it is important to note that these antibiotics were chosen based on the availability of urinary antibiotic concentrations/time profiles. Very few antibiotics have this information published, and most only have data describing the cumulative urinary antibiotic concentrations. Also, the data obtained to determine the urinary concentration/time profiles were primarily based on the clearance of healthy volunteers with no evidence of renal dysfunction or critical illness, which may limit the applicability of these results to other patient populations.

## Additional file

**Additional file 1: Table S1.** MIC distribution of enterobacteriaceae (n = 21) and *Pseudomonas aeruginosa* (n = 3) against ciprofloxacin, ceftriaxone, cefepime, and doripenem.

## Abbreviations

CLSI: Clinical and Laboratory Standards Institute
MICs: minimum inhibitory concentrations
MDROs: multidrug-resistant organisms
Cpk: peak concentration
T > MIC: percentage of time concentrations remained above MIC
PD: pharmacodynamics
PK: pharmacokinetics
TMP/SMX: trimethoprim/sulfamethoxazole
UTIs: urinary tract infections
Vd: volume of distribution
