# Supplementary material for: Rethinking urinary antibiotic breakpoints: analysis of urinary antibiotic concentrations to treat multidrug resistant organisms
Source: BMC Res Notes. 2018 Jul 20;11:497. doi: 10.1186/s13104-018-3599-8 (PMC6053836; doi:10.1186/s13104-018-3599-8)
Supplement: Supplementary file 1 — Additional file 1: Table S1. MIC distribution of enterobacteriaceae (n = 21) and Pseudomonas aeruginosa (n = 3) against ciprofloxacin, ceftriaxone, cefepime, and doripenem. [file 13104_2018_3599_MOESM1_ESM.docx]

Additional file 1: Table S1: MIC distribution of enterobacteriaceae (n=21) and *Pseudomonas aeruginosa* (n=3) against ciprofloxacin, ceftriaxone, cefepime, and doripenem

| Bacterial isolates and antibacterial agents | Percentage (%) of bacteria at each MIC value (µg/mL) | | | | | | | | | | | | |
| --- | --- | --- | --- | --- | --- | --- | --- | --- | --- | --- | --- | --- | --- |
|  | 0.016 | 0.032 | 0.064 | 0.125 | 0.25 | 0.5 | 1 | 2 | 4 | 8 | 16 | 32 | > 32 |
| Ciprofloxacin | | | | | | | | | | | | | |
| *E*. *coli* (n=10) |  | 10 |  |  |  |  | 10 |  |  |  |  | 80 |  |
| *E*. *cloacae* (n=3) |  |  |  |  |  |  |  |  |  |  |  | 100 |  |
| *P*. *mirabilis* (n=3) |  |  | 33 |  |  |  |  |  |  |  |  | 67 |  |
| *Citrobacter* sp. (n=2) | 50 |  |  |  |  |  |  |  |  |  |  | 50 |  |
| *K*. *pneumoniae* (n=1) |  |  |  |  |  |  |  |  |  |  |  | 100 |  |
| *Serratia* sp. (n=1) |  |  |  |  |  |  |  |  |  |  |  | 100 |  |
| *M*. *morganii* (n=1) |  |  |  |  |  |  |  |  |  |  |  | 100 |  |
| *P*. *aeruginosa* (n=3) |  |  |  | 67 |  | 33 |  |  |  |  |  |  |  |
| Ceftriaxone | | | | | | | | | | | | | |
| *E*. *coli* (n=10) |  |  | 40 | 40 |  |  |  |  |  | 10 |  |  | 10 |
| *E*. *cloacae* (n=3) |  |  |  |  |  |  |  |  |  |  |  |  | 100 |
| *P*. *mirabilis* (n=3) | 67 |  |  |  | 33 |  |  |  |  |  |  |  |  |
| *Citrobacter* sp. (n=2) |  |  |  | 50 |  |  |  |  |  |  |  |  | 50 |
| *K*. *pneumoniae* (n=1) |  |  |  |  |  |  |  |  |  |  |  | 100 |  |
| *Serratia* sp. (n=1) |  |  |  |  |  |  |  |  |  |  | 100 |  |  |
| *M*. *morganii* (n=1) | 100 |  |  |  |  |  |  |  |  |  |  |  |  |
| Cefepime | | | | | | | | | | | | | |
| *E*. *coli* (n=10) |  | 10 | 30 | 20 | 10 |  | 10 |  |  |  | 10 |  | 10 |
| *E*. *cloacae* (n=3) |  |  |  |  |  |  |  |  | 33 | 33 | 33 |  |  |
| *P*. *mirabilis* (n=3) |  |  |  | 67 | 33 |  |  |  |  |  |  |  |  |
| *Citrobacter* sp. (n=2) |  |  | 50 |  |  |  |  |  |  |  |  |  | 50 |
| *K*. *pneumoniae* (n=1) |  |  |  |  |  |  |  |  |  |  | 100 |  |  |
| *Serratia* sp. (n=1) |  |  |  |  |  | 100 |  |  |  |  |  |  |  |
| *M*. *morganii* (n=1) |  |  |  | 100 |  |  |  |  |  |  |  |  |  |
| *P*. *aeruginosa* (n=3) |  |  |  |  |  | 33 |  | 33 |  | 33 |  |  |  |
| Doripenem | | | | | | | | | | | | | |
| *E*. *coli* (n=10) |  | 90 | 10 |  |  |  |  |  |  |  |  |  |  |
| *E*. *cloacae* (n=3) |  |  |  |  |  |  |  |  |  |  |  |  | 100 |
| *P*. *mirabilis* (n=3) |  |  | 67 | 33 |  |  |  |  |  |  |  |  |  |
| *Citrobacter* sp. (n=2) |  | 50 |  |  |  |  |  |  |  |  |  | 50 |  |
| *K*. *pneumoniae* (n=1) |  |  |  |  |  | 100 |  |  |  |  |  |  |  |
| *Serratia* sp. (n=1) |  |  |  | 100 |  |  |  |  |  |  |  |  |  |
| *M*. *morganii* (n=1) |  |  | 100 |  |  |  |  |  |  |  |  |  |  |
| *P*. *aeruginosa* (n=3) |  |  |  |  | 67 |  |  | 33 |  |  |  |  |  |
| MIC was obtained via Etest® | | | | | | | | | | | | | |
